# Placental‐Derived Connective Tissue Matrix Mediates Murine Recurrent Laryngeal Nerve Regeneration

**DOI:** 10.1002/lary.70313

**Published:** 2025-12-22

**Authors:** Sunjay Anekal, Ananya Tadikonda, Gabriel Sobczak, Lena W. Chen, Aditya Bhatt, Troy Wesson, Patrick R. Finnegan, Stacey Halum

**Affiliations:** ^1^ Indiana University School of Medicine (IUSM) Indianapolis Indiana USA; ^2^ Department of Otolaryngology—Head and Neck Surgery IUSM Indianapolis Indiana USA

**Keywords:** cytokines, placentally derived connective tissue matrix, RLN, UVFP

## Abstract

**Objective:**

Unilateral vocal fold paralysis (UVFP) due to recurrent laryngeal nerve (RLN) injury is a common cause of dysphonia. No biotherapeutic injectable exists that directs laryngeal reinnervation after RLN injury. Placental‐derived connective tissue matrix (pd‐CTM) could fill this need, as it contains a plethora of cytokines with potential UVFP therapeutic benefits. This study aimed to identify and quantify the factors in a commercially available pd‐CTM (CTM Flow, CTM Biomedical, Lake Worth, Florida) and to study the effects of pd‐CTM on vocal fold microenvironment and glottic function in a mouse model of unilateral RLN injury.

**Methods:**

Cytokine expression (ng/mL) in pd‐CTM was characterized using a cytokine array and ELISA. In a separate experiment, C57/BL6 mice were divided into three groups: uninjured negative controls (*n* = 12), RLN transection with ipsilateral saline thyroarytenoid (TA) injection (*n* = 16), and RLN transection with ipsilateral pd‐CTM TA injection. Outcomes included laryngeal electromyography (L‐EMG) and video laryngoscopy after 7 and 28 days, with larynges then harvested and analyzed via immunohistochemistry (IHC) and qPCR.

**Results:**

pd‐CTM characterization showed moderate‐to‐high levels of neurotrophic (BDNF, CNTF, GDNF, NTF‐3), angiogenic (Angiogenin, VEGF‐D), tissue remodeling (bFGF, IGF‐1, HGF, TGF‐β3), and anti‐inflammatory factors (IL‐10, IL‐1Rα). L‐EMG demonstrated increased mean normalized area under the curve ratio in pd‐CTM treated mice compared to saline treated mice at the 28‐day time point indicating reinnervation (*p* < 0.001). IHC detected innervated neuromuscular junctions 28 days after pd‐CTM treatment.

**Conclusion:**

pd‐CTM may be a novel treatment option for patients with UVFP based on the neurotrophic, angiogenic, tissue remodeling, and anti‐inflammatory factors present.

**Level of Evidence:**

NA.

## Graphical abstract

Placentally derived connective tissue matrix (pd‐CTM) innately contains various neurotrophic, angiogenic, anti‐inflammatory, and tissue remodeling cytokines. In this mouse model of unilateral vocal fold paralysis, we investigate its effects on recurrent laryngeal nerve (RLN) regeneration and reinnervation of the right thyroarytenoid (TA) muscle after right RLN transection. Our findings suggest improvement in bilateral vocal fold stimulation, stimulated action potentials in the right TA muscle, and right TA muscle atrophy and reinnervation 28‐days post pd‐CTM treatment.

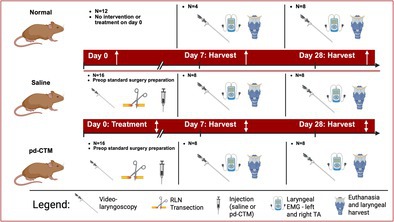

## 1 Introduction

Unilateral vocal fold paralysis (UVFP) is a common cause of voice disorders [1, 2, 3, 4]. With increases in non‐thyroid head and neck surgeries in recent years, iatrogenic injury of the recurrent laryngeal nerve (RLN) is one of the leading causes of UVFP [5]. Associated dysphonia is present in 97% of patients, negatively impacting communication. UVFP further diminishes quality of life due to morbidity from dysphagia and aspiration [4, 6, 7]. The combination of significant reductions in quality of life and loss of airway protection necessitates the prompt and robust treatment of UVFP.

Interventions within 12 months of onset include observation, voice therapy, and/or injection laryngoplasty [8, 9, 10]. Injectable options offer temporary benefits but do not promote laryngeal reinnervation (LR) or address the underlying RLN injury [10]. More invasive secondary procedures—repeat injections and/or permanent interventions such as medialization laryngoplasty (ML) with or without arytenoid adduction (AA) and LR—drive up healthcare utilization and costs [5, 8, 9, 10, 11, 12, 13]. Existing literature does not demonstrate significant differences between injection laryngoplasty, ML, AA, and LR when analyzing acoustic measures, laryngoscopy evaluation, and subjective voice outcomes; no intervention has been consistently shown to restore normal voicing [8, 9, 10, 11, 12, 13]. The challenges present in the current treatment algorithms for UVFP warrant investigation of an injectable with potential for long‐term glottic rehabilitation.

Several biomolecular pathways play important roles in reinnervation of the denervated larynx. Neurotrophic factors (BDNF, CNTF, NT‐3, and GDNF) are innately released from Schwann cells to promote neuronal regeneration and enhance neuronal survival [14, 15]. Additional growth factors that promote tissue remodeling (bFGF, IGF‐1), along with angiogenesis (VEGF‐A), prime the microenvironment around the injury site for neuronal proliferation and wound healing [16, 17, 18, 19]. Spontaneous reinnervation after RLN injury is common but may be aberrant, leading to synkinesis (uncoordinated activation of laryngeal muscles) with resultant dysphonia [20, 21]. Thus, therapeutic interventions that direct reinnervation and neuronal regeneration are of particular interest as a management strategy in UVFP.

Administration of neurotrophic factors in the larynx may direct reinnervation and mitigate synkinesis after UVFP and restore physiologic RLN function. Previous studies have found that exogenous administration of trophic factors such as bFGF, NT‐3, BDNF, GDNF, IGF‐1, and CNTF help promote organized RLN reinnervation of the larynx. Utilizing neurotrophic factors such as BDNF, GDNF, NT‐3, and CNTF to aid in RLN regeneration has also been explored in the literature [22, 23, 24, 25, 26, 27]. While placental derivatives have been shown to contain several neurotrophic (BDNF, NGF, NTF‐3, GDNF), angiogenic (Angiogenin, VEGF‐A, PDGF‐AA), tissue remodeling (IGF‐1, bFGF, HGF, TGF‐β3), and anti‐inflammatory factors (IL‐10, IL‐1Rα) [28, 29, 30, 31, 32, 33, 34, 35], no previous studies have investigated the use of placental derivatives for treatment of UVFP. Placentally derived connective tissue matrix (pd‐CTM) has shown promising results in orthopedic injuries and wound healing applications [31, 32, 33, 34]. CTM Flow is a type of commercially available pd‐CTM developed by CTM Biomedical for which relative protein expression and concentrations have not been previously characterized. We hypothesize that injecting pd‐CTM in the thyroarytenoid (TA) muscle ipsilateral to the transected RLN will promote neurogenesis, angiogenesis, and tissue remodeling leading to positive functional vocal fold outcomes in C57/BL6 mice. Specifically, this study aims to: (1) quantify the neurotrophic, angiogenic, and tissue remodeling factors in pd‐CTM using a cytokine array and ELISA; (2) measure early functional outcomes after pd‐CTM treatment of the denervated TA muscle through laryngeal electromyography (L‐EMG) and video laryngoscopy; and (3) assess microenvironmental changes within the TA muscle through immunohistochemistry (IHC) and quantitative polymerase chain reaction (qPCR).

## 2 Materials and Methods

### 2.1 pd‐CTM Characterization

Relative expression levels of 120 cytokines in a pd‐CTM (CTM Flow, CTM BioMedical, Lake Worth, Florida) were assessed through cytokine array analysis per manufacturer's protocol (C1000 Cytokine Array, AAH‐CYT‐1000‐2, RayBiotech, Peachtree Corners, Georgia). A Human VEGF‐A ELISA kit (Catalog# BMS277‐2, Thermo Fisher Scientific, Vienna, Austria) was used to determine the concentration (ng/mL) of VEGF‐A in pd‐CTM. The VEGF‐A concentration and the relative expression values of each cytokine were used to back calculate concentrations (ng/mL) of all detected cytokines in pd‐CTM (see Data S1).

### 2.2 Animal Model

**FIGURE 1:**
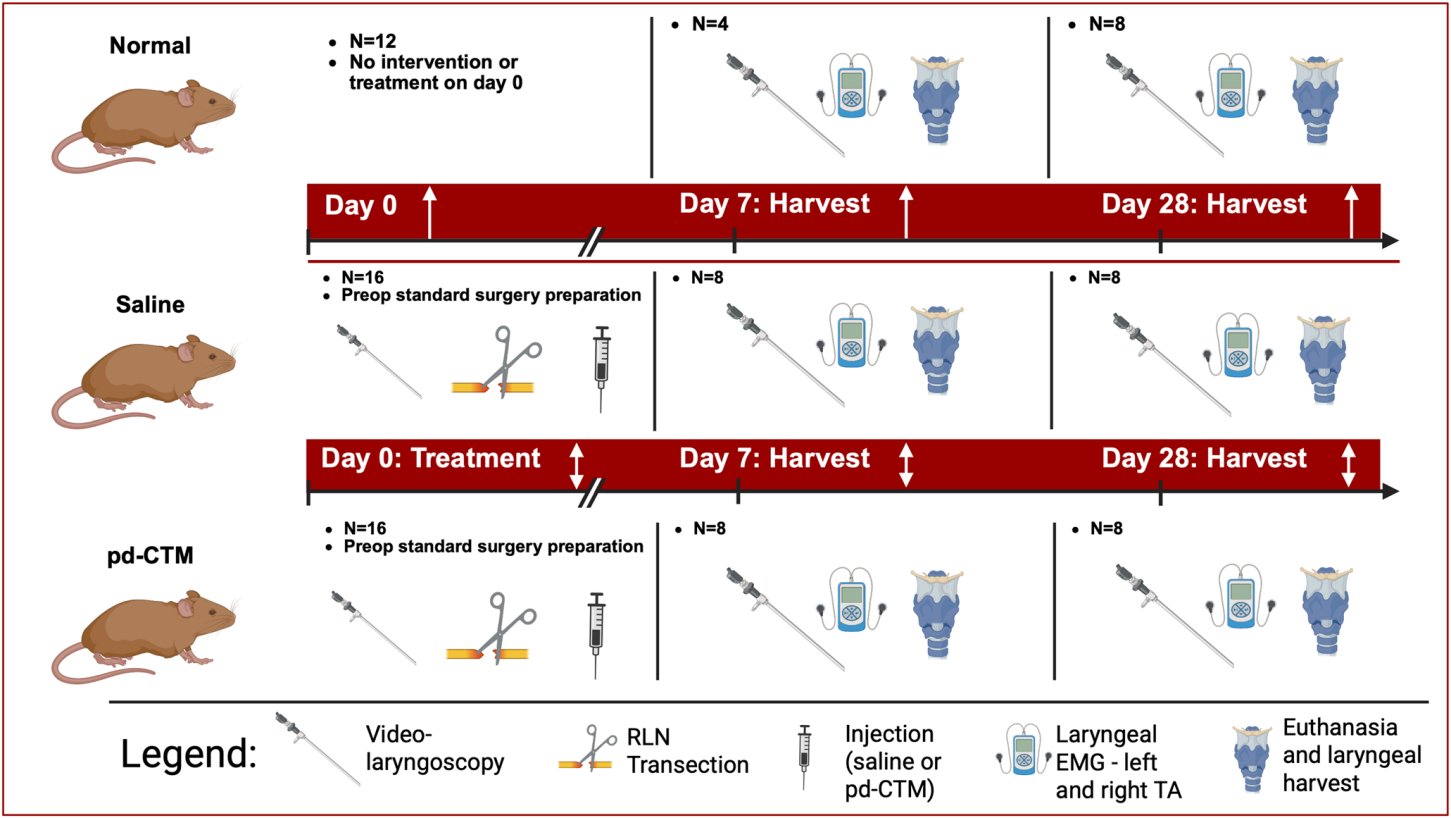
Experimental overview: Mice in the normal group underwent no intervention on Day 0. On Day 0, mice in the saline and pd‐CTM groups underwent video laryngoscopy, RLN transection, denervated thyroarytenoid muscle injection, and posttreatment videolaryngoscopy to confirm right vocal fold paralysis. Mice from all three groups underwent video laryngoscopy of vocal folds during bilateral RLN stimulation, bilateral TA EMG testing, euthanasia, and laryngeal harvest on either Day 7 or 28.

C57BL/6 mice were housed in the Laboratory Animal Resource Center (LARC) at Indiana University Indianapolis (IUI) and handled in accordance with the Indiana University Institutional Animal Care and Use Committee (IACUC) approved protocol 23150‐A4, with humane treatment of all animals. Mice were organized into three groups: untreated mice (uninjured normal; *n* = 12), unilateral RLN injury with saline injection into the TA muscle (injured control group; *n* = 16), and unilateral RLN injury with pd‐CTM injection into the TA muscle (injured treatment group; *n* = 16) (Figure 1). Survival surgery involving RLN transection took place at the start of the experiment (Day 0). At the 7‐ or 28‐day endpoint, the mice underwent videolaryngoscopy with RLN stimulation of the TA muscle, L‐EMG testing of the TA muscle, and euthanasia followed by laryngeal harvest (Figure 1).

### 2.3 RLN Injury and Treatment

Surgical procedures were all performed in the LARC facility at the Glick Eye Institute at IUSM. Mouse specific surgical instruments and a Steindorff Digital Video microscope (New York Microscope Company, Hickman, New York) were used for all surgeries. Ethiqa extended release (Ethiqa XR) at 3.25 mg/kg was administered to every mouse to reduce postoperative pain. Isoflurane was used for induction (4%) and maintenance (0%–2.5%) anesthesia. Videolaryngoscopy was used to evaluate vocal fold motion pre‐operatively. Surgical dissection of the submandibular glands and infrahyoid muscles was performed to expose and transect the right RLN (see Figure S1). After transection, 10 μL of pd‐CTM or 0.9% saline was injected into the right TA muscle. The infrahyoid muscles were sutured using a 6‐0 Vicryl suture. The ventral skin of the mice was closed using wound clips. Mice underwent videolaryngoscopy posttreatment to confirm right vocal fold immobility and were then allowed to recover from anesthesia (see Data S1).

### 2.4 Functional Outcomes

At Days 7 and 28, mice in their respective groups underwent harvest surgeries (Figure 1). Exposure of the larynx proceeded as in survival surgery until visualization of the larynx and the right and left RLNs were obtained. The mice underwent videolaryngoscopy using a flexible endoscope (Catalog# 624001000US, Ambu, Columbia, Maryland) and L‐EMG [UltraPro S100 EMG system (Catalog# 828‐060800, Natus, Middleton, Wisconsin)] of the TA muscles during bilateral RLN stimulation at 5.10 mAMP to assess vocal fold functional response. On the injured side, the RLN was stimulated both proximal and distal to the site of injury. The area under the curve (AUC) for each action potential waveform after right TA muscle stimulation was recorded and normalized to the AUC obtained after left TA muscle stimulation. Mean normalized AUC ratio was compared across all treatment groups. Following videolaryngoscopy and L‐EMG, euthanasia was performed in accordance with the IACUC protocol, and larynges were promptly harvested for immunohistochemistry and qPCR (Figure 1). Survival surgery videos were later reviewed in a blinded fashion, rating: (1) vocal fold motion upon stimulation, (2) vocal fold position at rest, and (3) the presence of any inflammation (see Data S1).

### 2.5 Immunohistochemistry and Histology

To ensure proper development of multiplex immunofluorescent assays, staining of positive and negative control C57BL/6 tissues was performed. Axial cross sections of larynges (5 μm) were stained using developed immunofluorescent multiplex assays for muscle atrophy with markers for muscle presence (Desmin) (Catalog# 73348S, CST, Danvers, MA) and atrophy (MURF1) (Catalog# 201941, Abcam, Waltham, MA), and for neuromuscular junction (NMJ) with markers for neuronal presence (NF‐L) (Catalog# C28E10, CST, Danvers, MA), acetylcholine receptor subunit (CHRNA1) (Catalog# 308307, Abcam, Waltham, MA), and synaptic vesicles (SV2) (Catalog# 317770, Abcam, Waltham, MA). Nuclei were stained with DAPI (3 μM‐5 min; Catalog #D21490, Invitrogen, Waltham, MA). A Leica DM2500 microscope was used at ×100, ×200, and ×400 magnification to obtain representative images of laryngeal cross sections (see Data S1).

### 2.6 qPCR

Two curls (20 μm each) were obtained from each tissue block. RNA was isolated using the RNeasy FFPE Mini kit (Catalog# 73504, Qiagen, Germantown, MD) to quantify expression of the following genes: *Ntf5*, *Bdnf*, *Ntf3* (neurotrophic genes), *Nos3* (angiogenic gene), and *Chrna1* [muscle‐type nicotinic acetylcholine receptor (nAchR) gene]. RT‐PCR was performed using the First Strand cDNA Synthesis kit (Catalog# NP100042; Origene Technologies Inc.; Rockville, MD). qPCR was performed using Origene Universal SYBR Green qPCR Master Mix (Catalog# NP100055) and Origene qSTAR Primer Pairs for *Chrna1* (Catalog# MP202479), *Bdnf* (Catalog# MP201391), *Ntf5* (Catalog# MP208867), *Nos3* (Catalog# MP208934), *Ntf3* (Catalog# MP208866), and *Gapdh* (Catalog# MP205604) (see Table S1). Analysis was done by calculating ΔΔC_*t*_ and determining fold changes (2^−ΔΔC^_*t*_) with *Gapdh* used as the reference gene (see Data S1).

### 2.7 Statistical Analysis

Statistical Package for the Social Sciences (SPSS) (IBM Inc.; Version 29.0.2.0, Armonk, NY) and RStudio (Posit Software, PBC, Version 4.4.3, Boston, MA) were used for statistical analysis on experimental data (see Data S1).

## 3 Results

### 3.1 pd‐CTM Characterization

**FIGURE 2:**
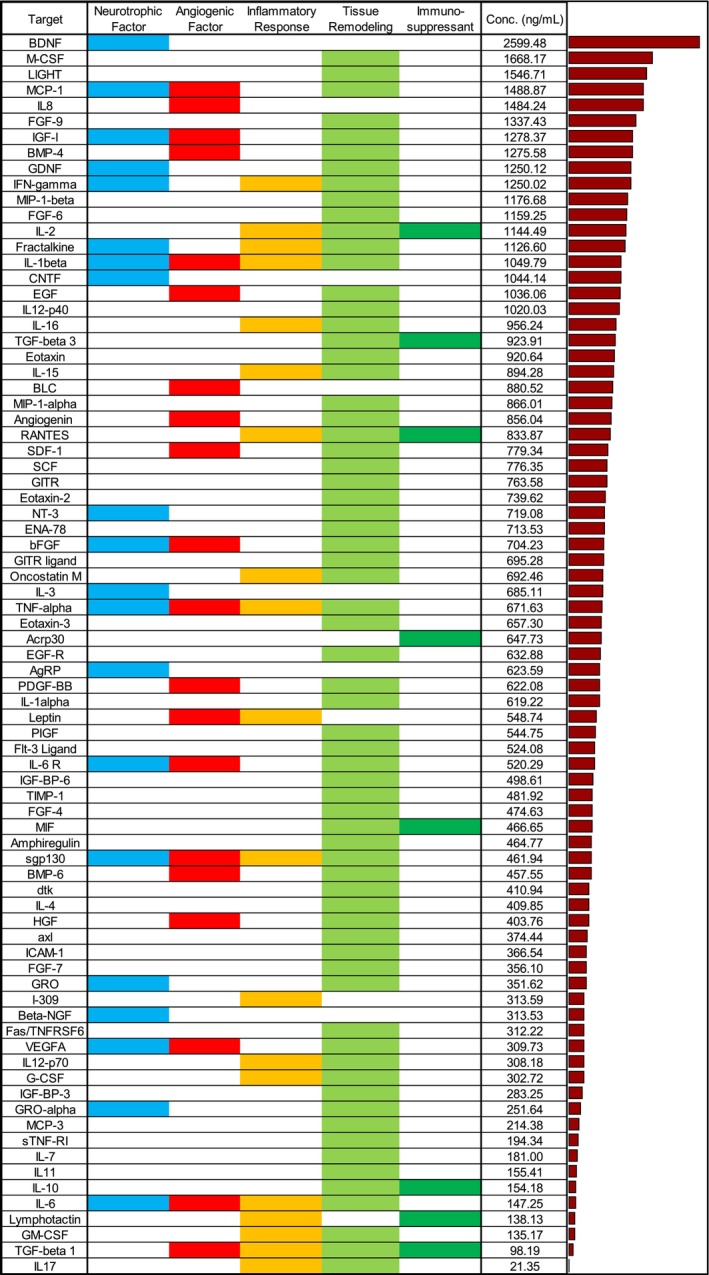
Concentrations (ng/mL) and properties (29 neurotrophic, 20 angiogenic, 19 anti‐inflammatory, 68 tissue remodeling, 8 immunosuppressant) of 79 select cytokines detected in pd‐CTM. These cytokines were found to have potential benefits in promoting a favorable TA muscle microenvironment after pd‐CTM injection along with immune compatibility of pd‐CTM as suggested by the presence of immunosuppressant factors.

120 unique cytokines were measured utilizing a combination of cytokine array and ELISA data. Figure 2 shows a breakdown of a subset of the cytokine array, highlighting 79 proteins with neurotrophic, angiogenic, anti‐inflammatory, tissue remodeling, and immuno‐suppressant properties. BDNF had the highest measured concentration amongst neurotrophic cytokines at 2599.5 ng/mL. Other neurotrophic cytokines, including GDNF, CNTF, and NT‐3, had moderate (349–875 ng/mL) to high (> 875 ng/mL) concentration levels in pd‐CTM. Tissue remodeling (e.g., IGF‐1, BMP‐4, FGF‐6, bFGF, TGF‐β3, HGF) and angiogenic (Angiogenin, PDGF‐BB) cytokines were found to have moderate to high concentrations (Figure 2).

### 3.2 Animal Model

Postoperatively, all but two animals had healthy appetites, normal weight gain, and showed no signs of distress. One mouse was wounded by its cage mates, and a second was euthanized because of apparent pain (hunching) 1 day following initial survival surgery. There were no problems with wound dehiscence or signs of infection in any animals.

### 3.3 Functional Outcomes

**FIGURE 3:**
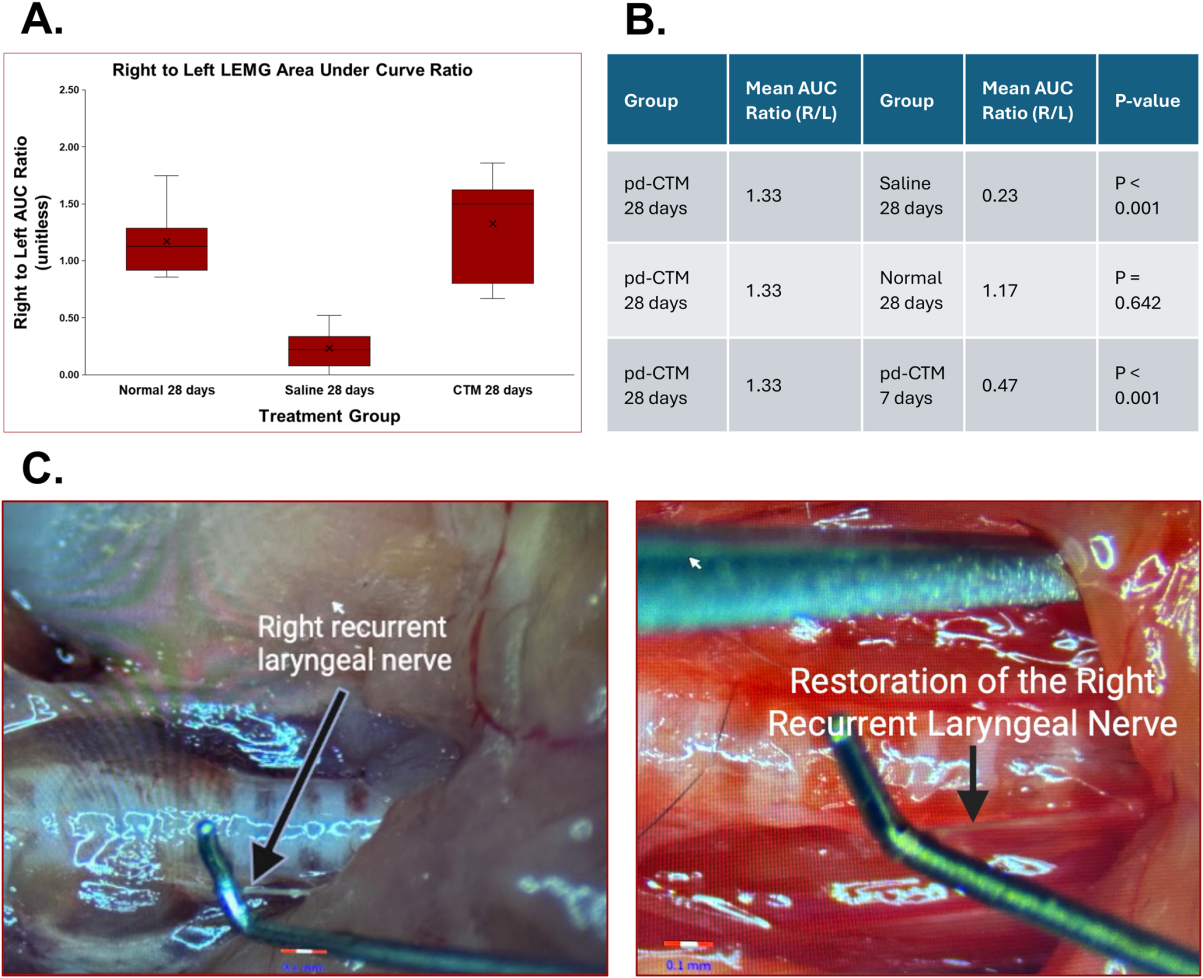
Assessment of neuronal structure and function after 28 days. The box‐and‐whisker plot (A) shows the mean normalized (right to left) L‐EMG area under the curve (AUC) ratios across the normal, saline, and pd‐CTM groups. (B) Comparison of mean AUC ratios of the pd‐CTM 28 group to the saline 28‐day, normal 28‐day, and pd‐CTM 7‐day groups, with *p* < 0.05 indicating a significant difference between the means. (C) Isolation of the right RLN at survival surgery (left) and 28 days post pd‐CTM treatment (right).

**FIGURE 4:**
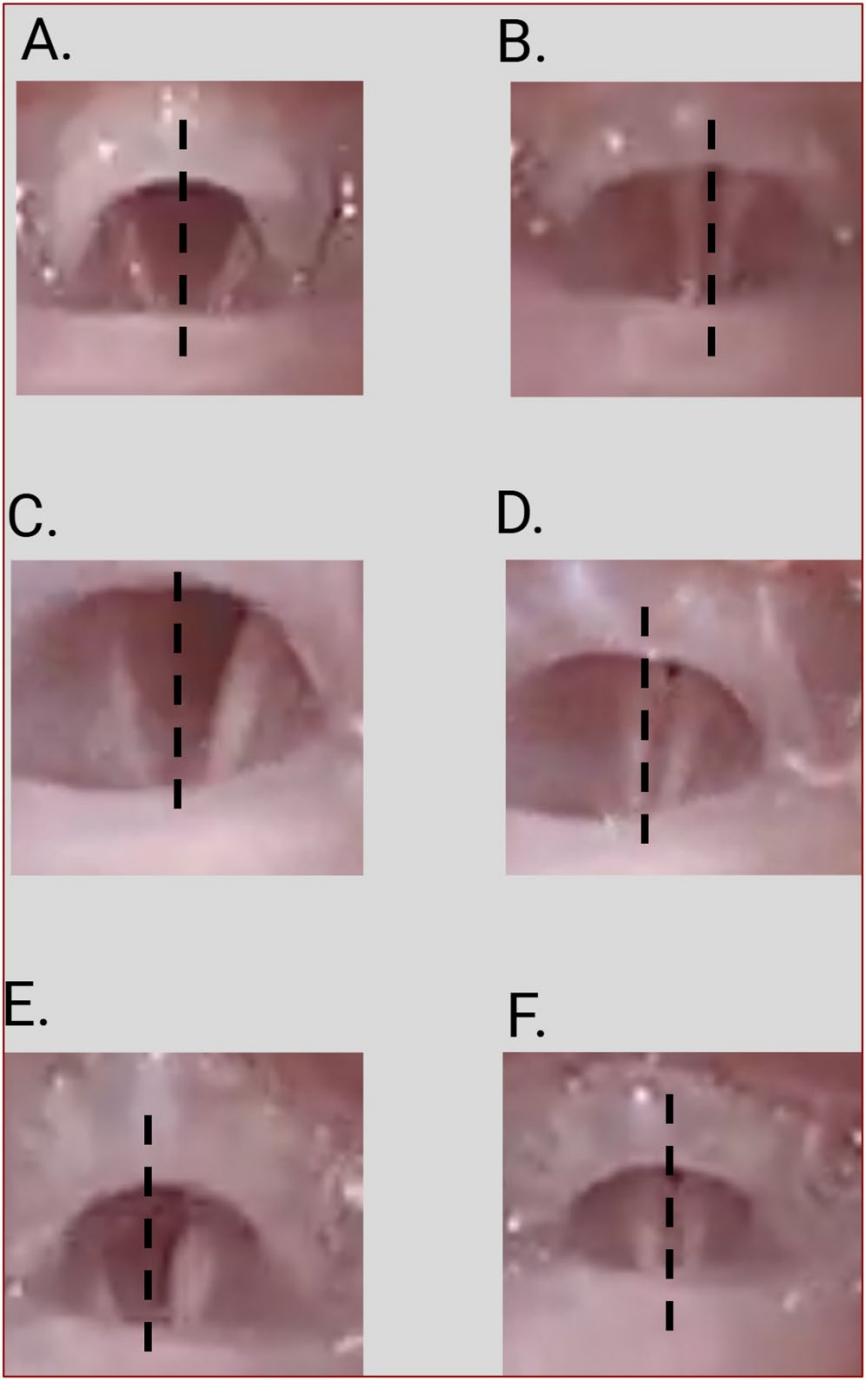
Videolaryngoscopy at 28 days. Representative images show vocal fold position in the normal (A and B), saline treated (C and D), and pd‐CTM (E and F) treated groups. (A, B) Baseline bilateral vocal fold motion in the normal 28‐day group that did not undergo transection. (C, D) Lateralized, immobile right vocal fold during inspiration and expiration in the saline 28‐day group. (E, F) Medialized vocal fold during inspiration and expiration in the pd‐CTM 28‐day group. Images on the left show vocal fold abduction during inspiration and images on the right show vocal fold adduction during expiration.

The mean normalized AUC ratio for the pd‐CTM 28‐day group was found to be 1.33 and was significantly higher than the saline 28‐day group mean of 0.23 (*p* < 0.001). Mean normalized AUC for the normal 28‐day group was found to be 1.17 with no significant difference in comparison to the pd‐CTM 28‐day group (*p* = 0.642) (Figure 3A). L‐EMG data showed a significant increase from the pd‐CTM 7‐day mean AUC ratio of 0.47 to the pd‐CTM 28‐day mean AUC ratio of 1.33 (*p* < 0.001). The significant improvement in mean AUC ratios in the pd‐CTM 28‐day group suggests restoration of right RLN activity in the TA muscle with pd‐CTM treatment compared to the saline treatment, along with improvement of right RLN activity in the TA muscle with pd‐CTM treatment over time (Figure 3B). No spontaneous vocal fold movement was visualized at the 7‐ and 28‐day time points in the pd‐CTM and saline groups. At 28 days, there was significant medialization in the pd‐CTM group compared to the saline group (*p* < 0.001) (Figure 4). At 28 days, bilateral stimulation showed significant left and right vocal fold response in the pd‐CTM (bilateral response in 7/8 mice, left response in 1/8 mice) and normal (response in 8/8 mice) groups compared to the saline group (bilateral response in 3/7 mice, left response in 4/7 mice) (*p* = 0.02). At 7 days, differences in bilateral stimulation response between the pd‐CTM and saline groups were found to be not significant (*p* = 0.398).

### 3.4 Immunohistochemistry

**FIGURE 5:**
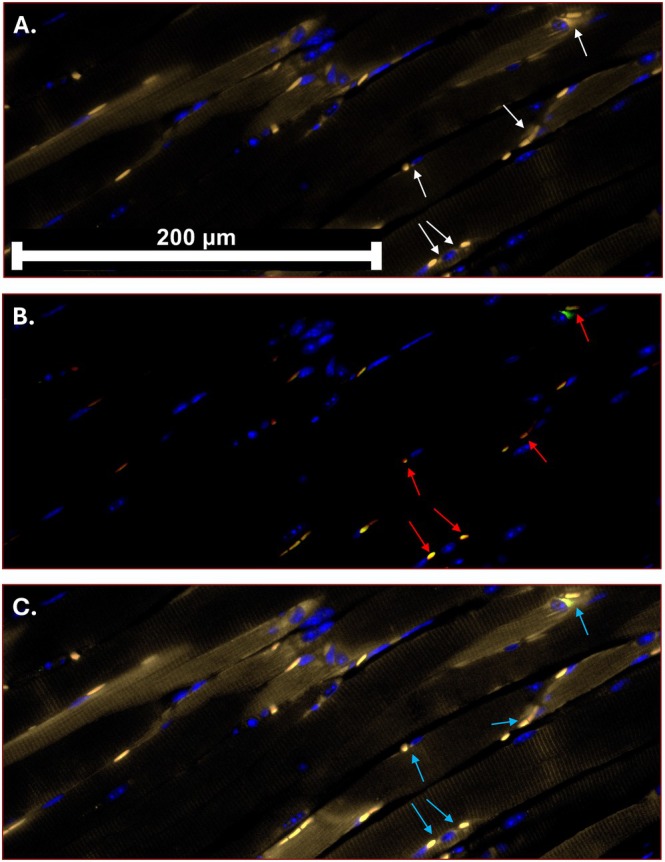
Representative images of nicotinic acetylcholine receptor (nAchR), neuronal, synaptic vesicle, and nuclear staining throughout the denervated TA muscle in the pd‐CTM 28‐day group. The arrows in (A) (white) identifies CHRNA1 staining (647 nm, yellow) at the nicotinic acetylcholine receptor (nAchR) as part of the motor endplate (MEP), and DAPI (blue) at the myofiber nucleus throughout the injured TA muscle. The arrows in (B) (red) point to SV2 (555 nm, red), NF‐L (455 nm, green), and DAPI combined staining throughout the injured TA muscle, indicating the presence of neurons at the same sites of nAchRs as shown in (A). The arrows in (C) (light blue) shows the overlay of stains in (A, B), indicating the presence of neurons and nAchRs at the same site throughout the treated TA muscle. Scale bar = 200 μm.

**FIGURE 6:**
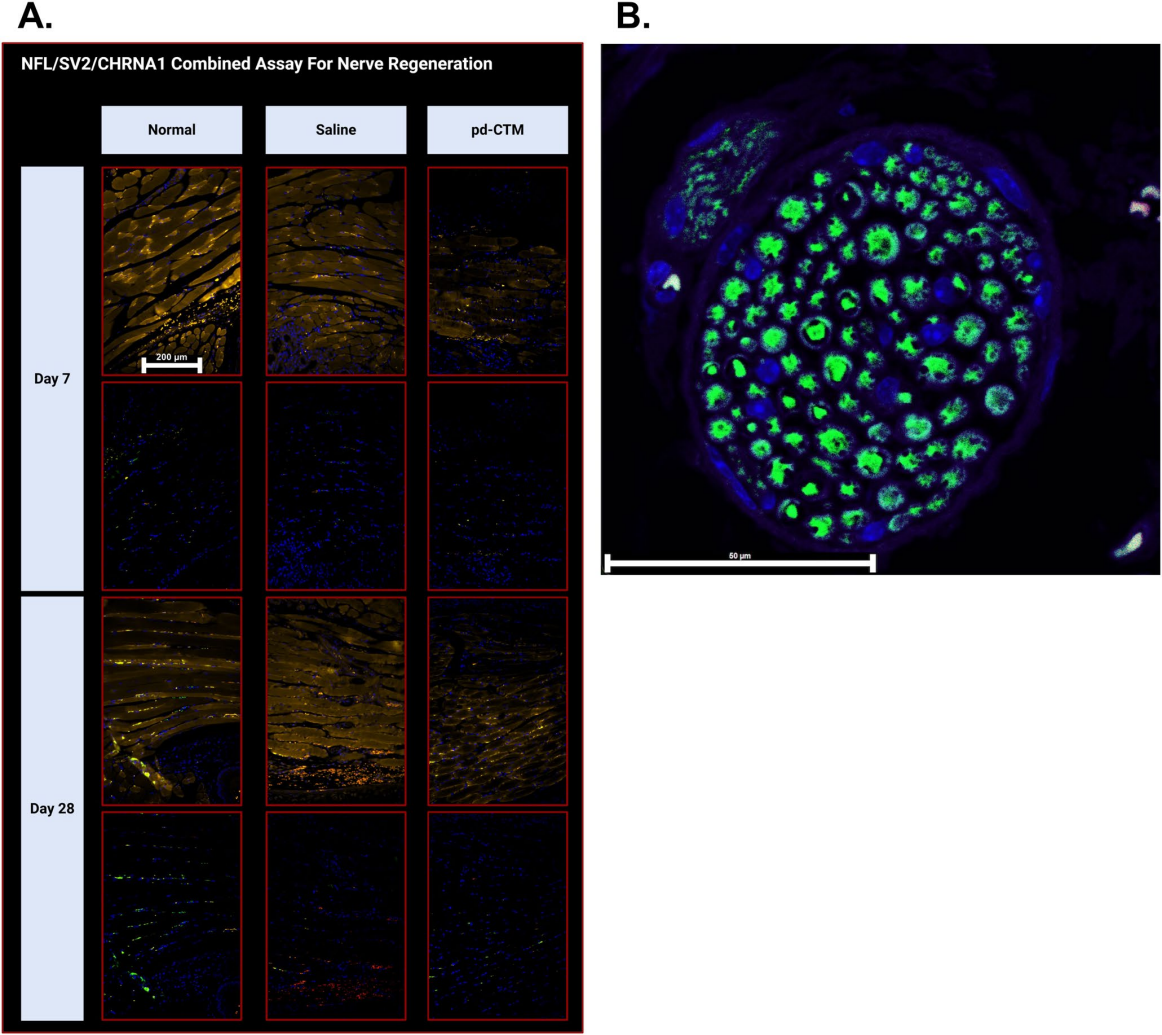
(A) Representative images showing CHRNA1 (yellow, top row), SV2 (red, bottom row), NF‐L (green, bottom row), and DAPI (blue, all images) in the right TA muscle (scale bar = 200 μm). Images are displayed for the 7‐ and 28‐day timepoints in the normal, saline, and pd‐CTM groups. CHRNA1 staining indicates presence of nAchRs throughout the TA muscle. SV2 and NF‐L combined stains show neuronal presence at the same site of nAchRs in the TA muscle. (B) NF‐L (green) staining of the right RLN, and DAPI (blue) nuclear staining, with the NF‐L positive regenerated axonal fibers spanning the site of RLN transection and entering the larynx. Scale bar = 50 μm.

**FIGURE 7:**
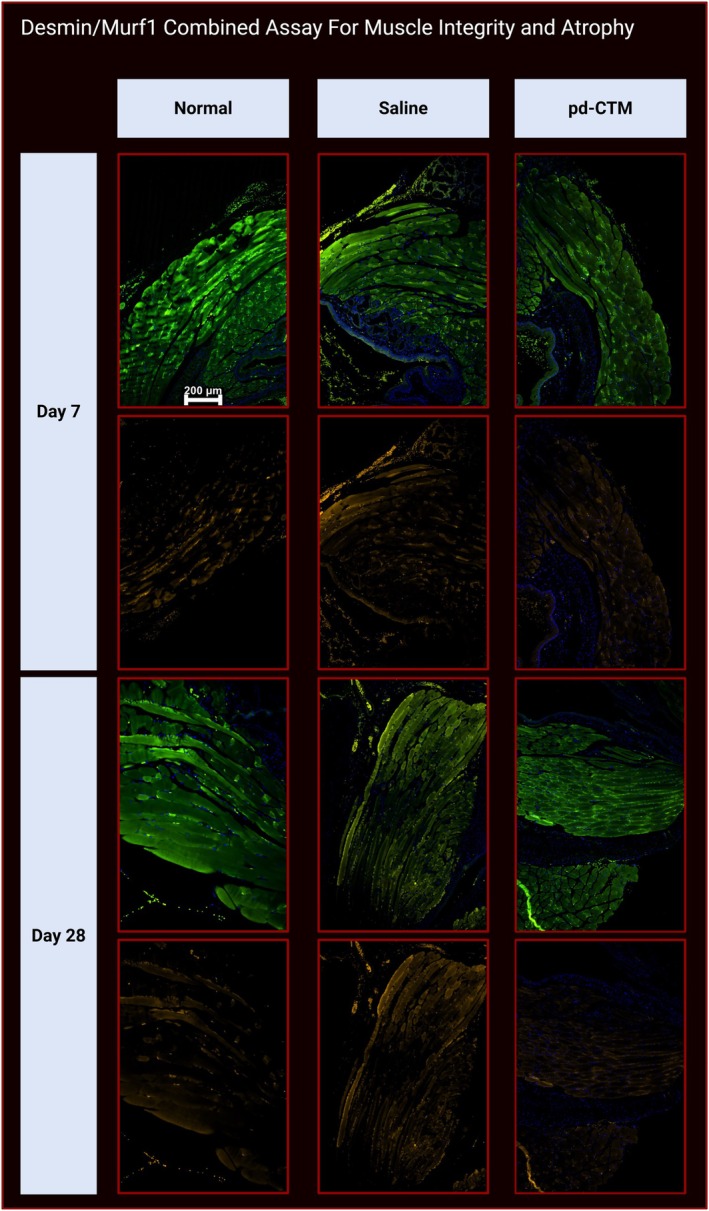
Desmin (488 nm, green, top row) staining at 7 and 28 days in the normal, saline, and pd‐CTM groups demarcates TA skeletal muscle fibers. MURF1 (555 nm, orange, bottom row) staining at 7 and 28 days across all three groups demonstrates comparable visible staining in the pd‐CTM and normal groups, and lower levels of staining in the pd‐CTM group relative to the saline group. These results highlight decreased TA muscle atrophy in the pd‐CTM, and normal groups compared to the saline group. Scale bar = 200 μm.

Figure 5 shows staining at motor endplates (MEPs) and neurons in the denervated TA muscle in the pd‐CTM 28‐day group. Assessment of the CHRNA1/SV2/NF‐L combined stains showed increased neuronal presence at the nAchRs in the pd‐CTM group relative to the saline group (Figure 6A). Beyond the RLN transection site, NF‐L and nuclear staining (DAPI) demonstrate intact right RLN fibers in the pd‐CTM 28‐day group (Figure 6B). Grossly, continuity between transected RLN endings appeared to be restored at 28 days after pd‐CTM treatment (Figure 3C), suggesting the RLN to be a source of laryngeal reinnervation. Minimal atrophy was visualized in the denervated TA muscle tissue of the pd‐CTM group compared to the saline group (Figure 7), indicating the positive impact of the various cytokines characterized in pd‐CTM on the TA muscle microenvironment.

### 3.5 qPCR

**FIGURE 8:**
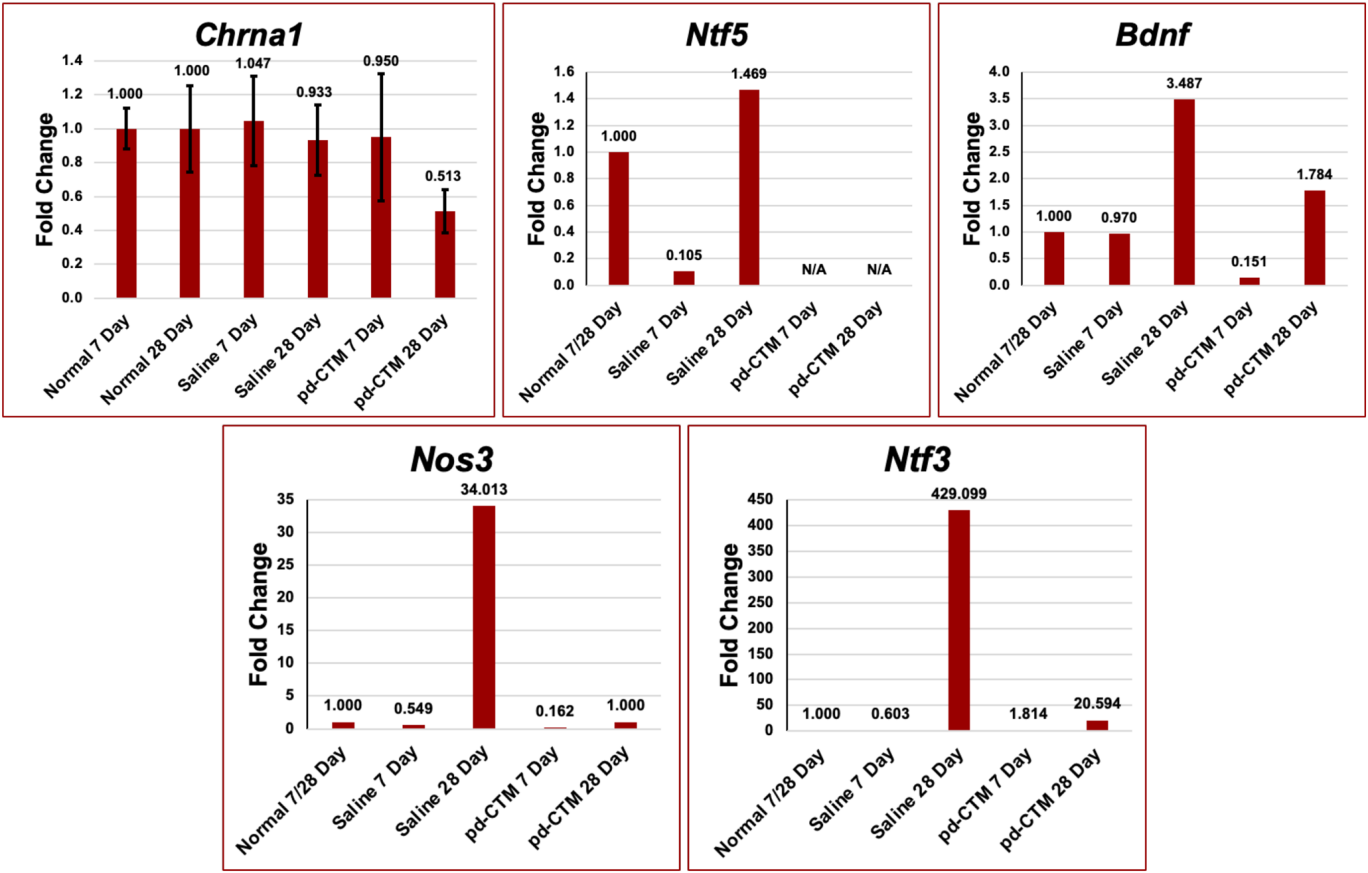
Expression levels of neurotrophic (*Ntf5*, *Bdnf*, *Ntf3*), angiogenic (*Nos3*), and motor endplate (*Chrna1*) genes in the treated (saline, pd‐CTM) and untreated (uninjured normal) TA muscles. Gene expression in the saline and pd‐CTM 7‐day groups was compared to the normal 7‐day group. Expression levels in the saline and pd‐CTM 28‐day groups were compared to the normal 28‐day group. No sample amplification was seen for *Ntf5* in pd‐CTM 7‐ and 28‐day groups.

Relative expression of neurotrophic (e.g., *Ntf5*, *Bdnf*, *Ntf3*), angiogenic (*Nos3*), and nAchR subunit (*Chrna1*) genes were detected across the 7‐ and 28‐day time points across all three experiment groups (Figure 8). At 7 days post treatment, the saline group showed increased gene expression of *Bdnf* and *Nos3* compared to the pd‐CTM treated group. While at the 28‐day time point, there was increased expression of *Bdnf*, *Nos3*, and *Ntf3* in the saline compared to the normal and pd‐CTM treated groups. No amplification occurred in the *Ntf5* pd‐CTM gene samples; thus, no comparative analysis was performed on this gene. The saline 7‐day group had a higher mean *Chrna1* expression value (1.047) compared to the normal (1.00) and pd‐CTM (0.950) 7‐day groups, with no statistical difference between the groups (*p* = 0.966). The saline group demonstrated higher *Chrna1* expression at the 28‐day time point than did the pd‐CTM group, but lower *Chrna1* expression when compared to the uninjured group; these differences were also not significant (*p* = 0.233).

## 4 Discussion

### 4.1 pd‐CTM Contains Therapeutic Cytokines

#### 4.1.1 A Novel Cytokine‐Based Therapy

While neurotrophic factors such as BDNF, GDNF, NGF, and NT‐3 have been previously detected in placentally derived biomaterials [28, 29], concentrations of such factors in placental products are not well established. By normalizing cytokine array data with an ELISA assay, pd‐CTM demonstrated moderate to high concentrations of neurotrophic, angiogenic, anti‐inflammatory, and tissue remodeling factors (Figure 2). Neurotrophic factors detected in pd‐CTM such as BDNF, GDNF, CNTF, NT‐3, VEGF‐A, IGF‐1 play a key role in axonal repair and neuronal regeneration [14, 15, 16, 17, 18]. Combination therapies with BDNF/GDNF and NT‐3/CNTF in nerve injury models have previously shown the benefits of administering multiple cytokines to promote nerve regeneration [14, 18, 24]. Thus, the inherent presence of neurotrophic factors coupled with tissue remodeling and angiogenic factors suggests that pd‐CTM could play a therapeutic role in selectively directing and promoting RLN reinnervation to the denervated TA.

#### 4.1.2 Clinical Relevance

Utilization of cytokine‐based therapies with injection laryngoplasty has been studied in the literature. Motor‐endplate expressing (MEE) cell delivery via collagen has demonstrated increased neurotrophic (*Bdnf*, *Chrna1*) gene expression, improved TA muscle volume, and increased normalized L‐EMG AUC [36]. Autologous fat grafts have been previously studied as a delivery medium for platelet rich fibrin (PRF) [37]. PRF contains numerous growth factors (VEGF, HGF, IGF, PDGF, TGF‐ß), and has been shown to improve phonation time and acoustic measures [37]. Given these promising results, additional studies that pair pd‐CTM with existing treatments are warranted.

### 4.2 pd‐CTM May Guide Early Reinnervation and Attenuate Atrophy

Functional outcomes and immunohistochemical findings in the current study suggest that pd‐CTM may help guide early TA reinnervation after RLN injury.

#### 4.2.1 Functional Outcomes

L‐EMG testing at 28 days showed increased mean normalized AUC in the pd‐CTM group compared to the saline group (Figure 3A). A significant increase in mean normalized AUC was also noted in the pd‐CTM 28‐day group compared to the pd‐CTM 7‐day group, suggesting improved injured RLN activity in the treated TA muscle over time [21, 38, 39]. The intraoperative finding of a regenerated and isolated right RLN (Figure 3D) in the pd‐CTM 28‐day group further supports that the L‐EMG findings were due to RLN regeneration directed to the TA muscle. While none of the paralyzed vocal folds had spontaneous motion due to the early analysis timepoint (28 days), the resting position of the pd‐CTM treated vocal folds differed, with more vocal folds resting in a medial position at 28 days in the pd‐CTM group compared to the saline group, suggesting pd‐CTM factors may have mitigated synkinetic reinnervation. No signs of laryngeal infection or vocal fold inflammation were present throughout the study, indicating the safety and immune compatibility of pd‐CTM in C57/BL6 mice.

#### 4.2.2 Immunohistochemical Findings

At the 28‐day time point, IHC with combined CHRNA1/NF‐L/SV2 staining showed diffuse neuronal and NMJ presence in the denervated pd‐CTM treated TA muscle, suggesting that exogenous administration of neurotrophic factors in the form of pd‐CTM supported neurogenesis and sustained nAchRs at the NMJs. Positive NF‐L and nuclear (DAPI) staining of the right RLN spanning the RLN transection site and entering the larynx (Figure 6B) supports the early regenerative potential of the neurotrophic cytokines in pd‐CTM. MURF1 staining showed visibly decreased muscle atrophy in the pd‐CTM group at both the 7‐ and 28‐day timepoints compared to the normal and saline groups. Across time, muscle atrophy in the pd‐CTM 28‐day group also showed markedly decreased atrophy (based on MURF1) relative to the pd‐CTM 7‐day group [40], suggesting attenuated atrophy coexisted with enhanced reinnervation, as would be expected. Overall, findings suggest that when pd‐CTM is delivered to the TA immediately upon RLN injury, the factors within pd‐CTM create a microenvironment that sustains muscle health and promotes early reinnervation.

### 4.3 Gene Expression Analysis

At 28 days, the pd‐CTM and normal groups showed lower levels of *Bdnf*, *Nos3*, and *Ntf3* gene expression than that of the saline controls (Figure 8). There was no significant difference in *Chrna1* expression at the 7‐ or 28‐day time point across groups. *Ntf5* gene expression was not detected in the pd‐CTM group at 7 or 28 days. As detected in the cytokine array, pd‐CTM contains numerous factors involved in neurogenesis and angiogenesis (Figure 2). Thus, lower neurotrophic gene expression in the pd‐CTM 28‐day group relative to the saline 28‐day group may suggest that exogenous administration of an injectate with a plethora of neurotrophic cytokines reduces endogenous TA muscle expression of genes involved in the production of such factors. Findings are consistent with previous literature describing negative feedback loops, with exogenous administration of substrates suppressing endogenous expression of genes and enzymes involved in the production pathway of that substrate [41, 42, 43].

### 4.4 Limitations

The current study is limited by the short follow‐up period and utilization of a small animal (mouse) model which may not be entirely clinically translatable. Tracking outcomes over additional timepoints in a variety of animal models will be important for future studies to better assess the microenvironmental changes that ensue over time after pd‐CTM treatment. Given synkinetic reinnervation of the posterior cricoarytenoid muscle (PCA) was not assessed, future studies should assess reinnervation markers (e.g., CHRNA1, SV2, NF‐L) of the PCA using IHC.

## 5 Conclusion

In this study, we present a novel implementation of a commercially available pd‐CTM in a mouse model of UVFP. The results, at an early follow‐up period of 28 days, suggest that pd‐CTM is a favorable injectable treatment for creating a microenvironment that promotes TA muscle reinnervation and function. Future studies will include longer time points and larger animal models of UVFP to fully elucidate the effects of pd‐CTM.

## Funding

This study was supported by the Department of Otolaryngology—Head and Neck Surgery, Indiana University School of Medicine CTM Biomedical provided funding and the commercially available placentally derived connective tissue matrix for testing and treatment in a C57/BL6 mouse model for this project.

## Conflicts of Interest

The authors declare no conflicts of interest.

## Supporting information


**Data S1:** lary70313‐sup‐0001‐supinfo.docx.

**Figure S1:** Isolated right recurrent laryngeal nerve (RLN).

**Table S1:** Forward and reverse sequences of each qPCR primer pair gene.

## Data Availability

The data that support the findings of this study are available on request from the corresponding author. The data are not publicly available due to privacy or ethical restrictions.
