# Supplementary material for: Placental‐Derived Connective Tissue Matrix Mediates Murine Recurrent Laryngeal Nerve Regeneration
Source: Laryngoscope. 2025 Dec 22;136(5):2220–31. doi: 10.1002/lary.70313 (PMC13067230; doi:10.1002/lary.70313)
Supplement: Supplementary file 1 — Data S1: lary70313‐sup‐0001‐supinfo.docx. [file LARY-136-2220-s001.docx]

**Supplemental Information**

**Methods**

**pd-CTM characterization**

Cytokine array analysis of pd-CTM (CTM Flow^TM^, CTM BioMedical, Lake Worth, Florida) was completed using the manufacturers protocol (C1000 Cytokine Array, AAH-CYT-1000-2, RayBiotech, Peachtree Corners, Georgia). Absolute concentration (ng/mL) of VEGF-A was acquired using a Human VEGF-A ELISA kit (Catalog# BMS277-2, Thermo Fisher Scientific, Vienna, Austria). The absolute concentration of every cytokine was acquired by taking the absolute concentration of VEGF-A and multiplying it by the ratio of relative concentration of VEGF-A to relative concentration of the respective cytokine. Concentration levels of cytokines in pd-CTM were divided into three groups: low, medium, and high concentration. Concentration ranges were determined by quartiles highlighting the 25^th^ and 75^th^ percentile values. Any cytokine below the 25^th^ percentile was categorized as having low concentrations, cytokines in between the 25^th^ and 75^th^ percentiles were categorized as having medium concentrations, and any cytokine found to have a concentration above the 75^th^ percentile was categorized as having high concentrations.

**Sterile Saline Preparation**

The 0.9% sterile saline solution used for saline injection was first prepared by dissolving 9 g of sodium chloride (NaCl) into 1000 mL of MiliQ water (CAS# 7732-18-5). Once this solution was thoroughly mixed, sterilization on the solution was performed using an autoclave. After sterilization, the saline was loaded into sterile 31-gauge insulin needles (Catalog# 0258217, BD Insulin Syringes with BD Ultra-Fine^™^ needle, Franklin Lakes, NJ) for injection.

**Survival Surgeries**

All surgeries were performed using a Kent Scientific mouse surgical kit (Kent Scientific Inc.; Torrington, CT). Mice were administered a subcutaneous injection of Ethiqa extended release (Ethiqa XR) 3.25 mg/kg and subsequently induced with isoflurane (4%) in an induction box. Once sedated, the mice were placed into a nose cone for maintenance of anesthesia (0-2.5%) in a supine position under a Steindorff Digital Video dissection microscope (New York Microscope Company, Hickman, New York). Abdomen and hands were secured to the surgical platform for stabilization. A flexible video laryngoscope (Catalog# 624001000US, Ambu, Columbia, Maryland) was inserted into the oropharynx to visualize baseline vocal fold motion. After visualization of the vocal folds, the ventral surface of the neck was shaved using an electrical razor. Sterilization with alternate washes of betadine and isopropyl alcohol was performed on the ventral surface of the neck. Draping of the surgical site was performed using sterile Glad Press ’N Seal plastic wrap. A single vertical, midline incision using a disposable 15-blade scalpel (GB0552, EXELINT^®^, Redondo Beach, CA) was made along the ventral surface of the neck to expose the submandibular glands. The submandibular glands were carefully divided using micro scissors to expose the infrahyoid muscles. The infrahyoid muscles were then sharply divided and lateralized to expose the pre-tracheal fascia, which was gently dissected until the tracheal rings were exposed. The right recurrent laryngeal nerve (RLN) was visualized between tracheal rings 4 and 6 (**Figure 1**) and was carefully dissected away from the inferior thyroid artery and trachea. The RLN was isolated using a micro dissecting hook (Item # 10064-14, Fine Science Tools Inc.; Foster City, CA). Once isolated, the RLN was transected sharply with micro scissors. A sterile 31g insulin needle was then used to inject a total of 10 µl of either sterile saline or pd-CTM into the thyroarytenoid (TA) muscle. Closure of the strap muscles was performed with 6-0 coated Vicryl sutures (Catalog# 103914 Ethicon^®^, Guaynabo, Puerto Rico). The skin was closed using a Reflex^™^ Clip System 7 mm wound clip applier (Catalog# 204-1000, Kent Scientific Inc.; Torrington, CT). Video laryngoscopy was subsequently performed to confirm immobility of the vocal fold ipsilateral to the transected recurrent laryngeal nerve. After emergence from anesthesia, the mice were returned to LARC facility cages with appropriate housing and post operative measures.

**Electromyography**

For intraoperative stimulation of the vocal folds, two Genuine Grass® 30-gauge reusable subdermal needle electrodes (F-E2-24, Natus, Middleton, Wisconsin) were connected to a handheld advanced stimulus probe and placed along the left and right RLN during bilateral vocal fold stimulation (5.10 mAMPs) with visualization through video laryngoscopy. Needles were placed proximal and distal to the site of RLN injury during TA muscle stimulation. During L-EMG measurements, an amplifier was connected to the following three platinum EMG needles: a ground (placed in the right hindlimb), a reference (placed in the pectoralis muscle ipsilateral to the nerve stimulated), and the third needle placed in the thyroarytenoid muscle ipsilateral to the nerve stimulated. Motor unit action potentials (MUAPs) were measured upon stimulation of both the right and left RLNs. Representative action potential responses were quantified by measuring the area under the curve (AUC). In each mouse, MUAPs measured on the right were normalized to MUAPs measured on the left to yield ratios of right to left MUAPs. These ratios were compared across all treatment groups in the same period (e.g., comparing pd-CTM to saline at the 28-day time point) and across different time frames within the same treatment group (e.g. comparing pd-CTM treated mice at 7 days to pd-CTM treated mice at 28 days).

**Videolaryngoscopy**

Videolaryngoscopy data was obtained using the Ambu Scope 5 Broncho 2.7/1.2 mm single use flexible endoscope and was stored on the Ambu tablet. Videos were organized by mouse with 6 videos per mouse: survival surgery pre-operation, survival surgery post-operation, harvest surgery pre-operation, harvest surgery intraoperative right RLN stimulation, harvest surgery intraoperative left RLN stimulation, and harvest surgery intraoperative bilateral RLN stimulation. Blinded analysis was completed on each video.

For vocal fold motion ratings: 0 indicated complete impairment, 1 indicated partial impairment, and 2 indicated no impairment. For vocal fold inflammatory rating: 0 indicated no inflammation, 1 indicated mild inflammation, and 2 indicated severe inflammation. For harvest surgery videos, preop vocal fold motion and inflammatory signs were recorded using the same grading system as the survival surgery videos. Response sidedness during bilateral stimulation was evaluated with the following coding system: 0 indicating no stimulation of either side, 1 indicating only right-side stimulation, 2 indicating only left side stimulation, and 3 indicating bilateral stimulation.

**IHC Assay Development and Laryngeal Tissue Staining**

Laryngeal Tissue Processing and Staining Once larynges were harvested from the experimental mice, they were placed in 10% NBF for 24 hours with gentle oscillation. After this 24-hour period, the larynges were placed into warm HistoGel (Catalog# HG-4000-012, Richard-Allan Scientific, Kalamazoo, Michigan) and positioned at a 10-degree posterior tilt. Once allowed to cool, larynges were sectioned into 0.5 cm blocks in solid HistoGel and sent to the IU Indianapolis Histology Core. Axial sections of the larynges were obtained (5 µm) and stained according to the IHC protocol developed in our lab.

IHC Assay Development Assay development on positive and negative control tissue was first conducted to develop appropriate laryngeal tissue staining protocol. For the Desmin/MURF1 and NF-L/CHRNA1/SV2 combinations, directed conjugated immunofluorescence (IF) detection was performed on mouse skeletal muscle (positive control), mouse large intestine (negative control), and untreated mouse larynx (test tissue). After appropriate immunopositivity was visualized, experimental laryngeal tissues were stained. Working concentrations of each individual antibody in Desmin/MURF1 and NF-L/CHRNA1/SV2 were 1 µg/mL. Wash buffer was administered with 1x Tris Buffered Saline 3 times after each reagent except DAPI. After DAPI counterstain, slides were rinsed in MilliQ water and then coverslipped. Protocol in brief: Slides were first deparaffinized in 60C for 15-30 minutes, followed by consecutive washes in xylene, ethanol, and MiliQ water. High pH antigen retrieval took place for 30 minutes. Leica Novolink protein block was applied to the tissue for 10 minutes. Desmin/MURF1 cocktail and NF-L/CHRNA1/SV2 cocktail was added to their respective slides for 30 minutes, followed by a 3 mM DAPI counter stain for 3 minutes. Slides were cover slipped and viewed using a Leica DM2500 microscope. The regenerated right RLN beyond the level of transection in the pd-CTM 28-day group was imaged using the Leica TCS SP8 Dive confocal/multiphoton microscope at the Indiana Center for Biological Microscopy.

**qPCR**

The forward and reverse sequences for the Origene qSTAR Primer Pairs are shown in **Table 1**. For samples prepared for *Chrna1* expression analysis, one cDNA sample was obtained from each mouse in each treatment group and was added to the qPCR plate. *Bdnf*, *Nos3*, *Ntf3*, and *Ntf5* samples were obtained from each animal and pooled into their respective treatment groups (e.g., cDNA sample from each pd-CTM 28-day mouse was pooled into one sample). Each pooled sample was then added in triplicate (technical replicates) to the qPCR plate and was run on the Applied Biosystems ViiA7 Realtime Thermal Cycler to obtain cycle threshold (CT) values.

**Statistical Analysis**

The Shapiro-Wilks test was conducted on numerical data (e.g., L-EMG, qPCR, myofiber diameter) to assess normality within each experimental group. The interquartile test using Microsoft Excel (Microsoft, Inc.; Version 16.92, Redmond, WA) (nonparametric data) and Grubbs test using RStudio (parametric data) were conducted to exclude outlier data points on numerical data. A one-way ANOVA with or without Tukey HSD was conducted on numerical data comparing different treatment groups at the same time point (e.g., Saline 28-day vs. pd-CTM 28-day). Independent samples T-test was conducted on numerical data comparing mice receiving the same treatment at different time points (e.g., pd-CTM 7-day vs 28-days). Chi-square analysis was performed on categorical data (e.g., videolaryngoscopy data) to compare vocal fold motion, stimulation, and position at different time points and across different groups.

**Results**

**Videolaryngoscopy**

Comparison of vocal fold inflammation (induration, erythema, fold irregularity) showed significant improvement at day 7 compared to day 0 in the pd-CTM (p = 0.046) and saline (p = 0.046) groups. Decreased vocal fold inflammation was also noted at 28 days in the pd-CTM (p = 0.008) and saline (p = 0.014) groups compared to the day of survival surgery (day 0).

**Supplemental Tables**

| Gene | Primer Catalog Number | Forward/Reverse | Sequence |
| --- | --- | --- | --- |
| *Chrna1* | 202479 | Forward | CTTAACCAGCCTGGTGTTCTACC |
|  |  | Reverse | GCTCCACAATGACCAGAAGGAAC |
| *Bdnf* | 201391 | Forward | GGCTGACACTTTTGAGCACGTC |
|  |  | Reverse | CTCCAAAGGCACTTGACTGCTG |
| *Ntf5* | 208867 | Forward | CCTGCGTCAGTACTTCTTCGAG |
|  |  | Reverse | GCCTTGCATTCTGAGAGCCAGT |
| *Nos3* | 208934 | Forward | CGCAAGAGGAAGGAGTCTAGCA |
|  |  | Reverse | TCGAGCAAAGGCACAGAAGTGG |
| *Ntf3* | 208866 | Forward | CTACTACGGCAACAGAGACGCT |
|  |  | Reverse | GGTGAGGTTCTATTGGCTACCAC |
| *Gapdh* | 205604 | Forward | CATCACTGCCACCCAGAAGACTG |
|  |  | Reverse | ATGCCAGTGAGCTTCCCGTTCAG |

**Table 1:** Forward and reverse sequences of each qPCR primer pair gene

**Supplemental Figures**


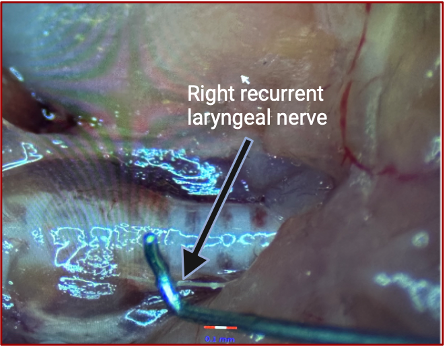


**Figure 1:** Isolated right recurrent laryngeal nerve (RLN)
