# Supplementary figures and images for: Placental‐Derived Connective Tissue Matrix Mediates Murine Recurrent Laryngeal Nerve Regeneration
Source: Laryngoscope. 2025 Dec 22;136(5):2220–31. doi: 10.1002/lary.70313 (PMC13067230; doi:10.1002/lary.70313)

# Supplemental Figure 1

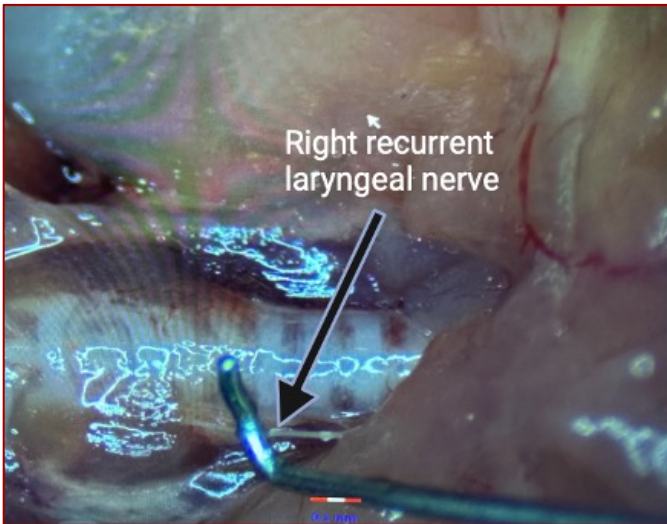

Supplement: Supplementary file 2 — Figure S1: Isolated right recurrent laryngeal nerve (RLN). [file LARY-136-2220-s002.pdf]
