# Supplementary material for: Placental‐Derived Connective Tissue Matrix Mediates Murine Recurrent Laryngeal Nerve Regeneration
Source: Laryngoscope. 2025 Dec 22;136(5):2220–31. doi: 10.1002/lary.70313 (PMC13067230; doi:10.1002/lary.70313)
Supplement: Supplementary file 3 — Table S1: Forward and reverse sequences of each qPCR primer pair gene. [file LARY-136-2220-s003.docx]

**Supplemental Tables**

| Gene | Primer Catalog Number | Forward/Reverse | Sequence |
| --- | --- | --- | --- |
| *Chrna1* | 202479 | Forward | CTTAACCAGCCTGGTGTTCTACC |
|  |  | Reverse | GCTCCACAATGACCAGAAGGAAC |
| *Bdnf* | 201391 | Forward | GGCTGACACTTTTGAGCACGTC |
|  |  | Reverse | CTCCAAAGGCACTTGACTGCTG |
| *Ntf5* | 208867 | Forward | CCTGCGTCAGTACTTCTTCGAG |
|  |  | Reverse | GCCTTGCATTCTGAGAGCCAGT |
| *Nos3* | 208934 | Forward | CGCAAGAGGAAGGAGTCTAGCA |
|  |  | Reverse | TCGAGCAAAGGCACAGAAGTGG |
| *Ntf3* | 208866 | Forward | CTACTACGGCAACAGAGACGCT |
|  |  | Reverse | GGTGAGGTTCTATTGGCTACCAC |
| *Gapdh* | 205604 | Forward | CATCACTGCCACCCAGAAGACTG |
|  |  | Reverse | ATGCCAGTGAGCTTCCCGTTCAG |

**Table 1:** Forward and reverse sequences of each qPCR primer pair gene
